# Impact of intravenous dexmedetomidine on gastrointestinal function recovery after laparoscopic hysteromyomectomy: a randomized clinical trial

**DOI:** 10.1038/s41598-022-18729-0

**Published:** 2022-08-27

**Authors:** Yu Wu, Zenghua Cai, Lishuang Liu, Jinbao Wang, Yanli Li, Yuling Kang, Ni An

**Affiliations:** 1 Department of Anesthesiology, Bethune International Peace Hospital, Shijiazhuang, 050082 China; 2 Department of Obstetrics and Gynecology, Bethune International Peace Hospital, Shijiazhuang, 050082 China; 3 Department of Anesthesiology and Pain, Troop 32295 of the Chinese People’s Liberation Army, Liaoyang, China

**Keywords:** Gastrointestinal system, Clinical pharmacology, Randomized controlled trials

## Abstract

Postoperative intestinal ileus is common after laparoscopic surgery, the incidence of those after hysterectomy was 9.2%. Anesthesia is one of the independent risk factors of postoperative ileus. Dexmedetomidine has been widely used in perioperative anesthesia and previous reports suggested that intraoperative dexmedetomidine may be associated with the improvement of gastrointestinal function recovery after abdominal surgery. We hypothesized that dexmedetomidine could improve gastrointestinal function recovery after laparoscopic hysteromyomectomy. Participants in elective laparoscopic hysteromyomectomy were enrolled with a single dose of 0.5 μg kg^−1^ dexmedetomidine or the same volume of placebo intravenously administered for 15 min, followed by continuous pumping of 0.2 μg kg^−1^ h^−1^ of corresponding drugs until 30 min before the end of surgery. The primary outcome was the time to first flatus. Secondary outcomes were the time to first oral feeding and the first defecation, the occurrence of flatulence, pain score and postoperative nausea and vomiting until 48 h after the surgery. Eventually, 106 participants (54 in dexmedetomidine group and 52 in placebo group) were included for final analysis. The time to first flatus (SD, 25.83 [4.18] vs 27.67 [3.77], P = 0.019), oral feeding time (SD, 27.29 [4.40] vs 28.92 [3.82], P = 0.044), the time to first defecation (SD, 59.82 [10.49] vs 63.89 [7.71], P = 0.025), abdominal distension (n%, 12 (22.2) vs 21 (40.4), P = 0.044), PONV at 24 h (n%, 10 (18.5) vs 19 (36.5), P = 0.037), NRS 6 h (3.15(0.68) vs 3.46 (0.87), P = 0.043) and NRS 12 h (3.43 (0.88) vs 3.85 (0.85), P = 0.014) of dexmedetomidine group were significantly shorter than those of the placebo group. Intraoperative dexmedetomidine reduced the time to first flatus, first oral feeding, and first defecation. These results suggested that this treatment may be a feasible strategy for improving postoperative gastrointestinal function recovery in patients undergoing laparoscopic hysteromyomectomy.

## Introduction

The temporary suppression of gastrointestinal motility after surgery is called postoperative ileus (POI), with clinical manifestations of nausea, vomiting, abdominal distention, oral intolerance, and insufficient flatus and defecation. Inhibition of gastrointestinal motility immediately after surgery is mainly caused by anesthetics, opioid analgesics and the surgery itself^1^. Moreover, intraoperative bowel manipulation activates inhibitory neuronal reflexes involving both adrenergic and non-adrenergic pathways and leads to intestinal edema due to excessive intravascular fluid load^2,3^. The incidence of POI is 10–30%, which is related to the type of surgery and the site of resection^4,5^. Laparoscopic hysteromyomectomy is one of the most common gynecological procedures in the world, due to its well-known advantages such as less pain, less bleeding, and shorter hospital stays^6^. The incidence of POI after hysterectomy was 9.2%, and one of the independent risk factors of POI is anesthesia^7^. Sheyn et al. concluded that approximately 0.12–1.1% of patients who underwent hysterectomy developed small bowel obstruction^8^, which is the most severe subtype of POI and requires surgical intervention. Increased incidence of postoperative nausea and vomiting (PONV) by POI is associated with a longer hospital stay, worsening patient condition, increased 30-day readmission rates and higher hospital costs^9,10^.

Pharmacologic treatment of POI remains problematic as most agents are unreliable and unsubstantiated with robust clinical trials^11,12^. Dexmedetomidine is a highly selective α2 adrenergic receptor agonist with sedative, analgesic, anti-sympathetic and anti-anxiety effects, and has been widely used in perioperative anesthesia and intensive care units^13^. Alpha 2 receptors are mainly distributed in the presynaptic membrane upon the postsynaptic membrane of the central nervous system, and also in the intestinal smooth muscle cell membrane surface^14^. Gastrointestinal peristalsis relies mainly on the stimulation of the parasympathetic nerve and the inhibition of the sympathetic nerve^15^. The majority studies of dexmedetomidine on bowel function have been conducted in patients undergoing gastrointestinal surgery^16^. A previous study focusing on abdominal hysterectomy used postoperative pain scores as the primary outcome, suggesting that dexmedetomidine was able to reduce the scores, but had no effect on bowel function^17^. Another study of dexmedetomidine for postoperative analgesia in gynecological laparoscopic surgery had pain scores as the primary outcome showed that postoperative analgesia with dexmedetomidine compared to opioids could reduce the time to recovery of bowel function^18^. However, to the best of our knowledge, the effect of dexmedetomidine on gastrointestinal function recovery after laparoscopic hysteromyomectomy has not been researched. Thus, we designed this prospective double-blind, placebo-controlled randomized clinical trial to verify our hypothesis that intraoperative administration of low-dose dexmedetomidine intravenously could promote the postoperative recovery of gastrointestinal function.

## Methods

### Study design

The double-blind, placebo-controlled randomized clinical trial study enrolled patients who underwent laparoscopic hysteromyomectomy from November 1, 2021, to January 1, 2022. This study was approved by the Ethics Committee of Bethune International Peace Hospital (No. 2021-KY-154) and registered at the Chinese Clinical Trial Registry (ChiCTR2100052392. registered on 24/10/2021). All participants gave written informed consent. This study followed the Uniform Standard for Reporting Randomized Clinical Trials (CONSORT) reporting guidelines. Inclusion criteria: patients of the American Society of Anesthesiologists (ASA) grade I–II, aged 40–65 years old who were scheduled to undergo elective laparoscopic hysteromyomectomy by the same gynecologist. Exclusion criteria: stress ulceration, ileus, bacterial translocation, intra-abdominal hypertension, abdominal compartment syndrome, previous abdominal surgery, severe liver and kidney dysfunction, secondary or tertiary heart block, slow arrhythmia of baseline heart rate below 50 times/minute, cognitive dysfunction, history of difficult airway or delayed extubation, opioid abuse, allergy for dexmedetomidine and other anesthetics, preoperative gastrointestinal bleeding (> 100 mL), emergency surgery.

### Randomization and blinding

Study statisticians were not involved in the recruitment or drug provision of patients. Eligible participants were randomized in a 1:1 ratio to receive dexmedetomidine or saline placebo during surgery. The random sequence is a computer-generated random number based on the network security system. A nurse not involved in the study filled the corresponding drugs into identical 50 ml syringes, the information of which was contained in a sequentially numbered sealed envelope. Patients themselves, the gynecologist, and the researchers evaluating the results were all unaware of the specific grouping.

### Sample size

The sample size was calculated based on the time to first flatus by a online statistical computing system. Biostatistics team of CMT. URL https://www.biostats.cn/statbox/. According to the pilot study, the time to first flatus was equivalent to 27 (3.28) in the control group and 25 (2.94) in the dexmedetomidine group. Assume that the ratio of the two groups is 1:1, using a two-sided test with a significance level (α) of 0.05 and a power (1 − β) of 0.90, and the required sample size was 52 in each group. Considering a 15% withdrawal and loss for follow-up rate, 120 patients were included in this study.

### Anesthesia procedure

Standard monitoring including electrocardiogram, heart rate, noninvasive blood pressure and pulse oxygen saturation was initiated upon arrival in the operating room. A single dose of 0.5 μg kg^−1^ dexmedetomidine or the same volume of placebo (normal saline) was intravenously administered in 15 min, followed by continuous pumping of 0.2 μg kg^−1^ h^−1^ of corresponding drugs until 30 min before the end of surgery. 15 min after the beginning of infusion, intravenous induction was achieved by midazolam 0.02–0.04 mg kg^−1^, cisatracurium 0.2 mg kg^−1^, sufentanil 0.2–0.3 μg kg^−1^, and propofol 1.0–2.5 mg kg^−1^. After endotracheal intubation, volume-controlled ventilation was performed according to standard weight (7 ml kg^−1^). The end-tidal CO_2_ pressure (PETCO_2_) should be between 35 and 45 mmHg. Bispectral Index (BIS) was maintained at 40–60 by continuously intravenous injection of propofol 50–100 μg kg^−1^ min^−1^ and remifentanil 0.1–1.0 μg kg^−1^ min^−1^, and the neuromuscular block was maintained by cisatracurium 1–2 μg kg^−1^ min^−1^. 10 mg dolasetron was administered intravenously to prevent PONV. Atropine 0.5 mg was given when the patient’s heart rate was below 50 beats per minute. Urapidil 5 mg or ephedrine 6 mg was given when the patient’s blood pressure was increased or decreased by over 20% of baseline, respectively. By the end of the epidermal suture, a mixture of sufentanil 5 μg, neostigmine 1 mg and atropine 0.5 mg was administered intravenously. The postoperative analgesic regimen consisted of regular intravenous administered parecoxib 40 mg every 12 h, and tramadol 100 mg for oral administration when the numeric rating scale (NRS) exceeded 3.

### Outcome measures

The primary outcome was the time (hour) of the patient’s first flatus from the end of surgery. The secondary outcomes included the time to the first defecation and first oral feeding, pain score and PONV. NRS was used for pain evaluation^19^, and 10-point Likert scale for the severity of PONV^20^. Data were collected through interviews with patients by trained researchers who were double-blinded to the study protocol. The doctors involved in the study were not involved in data collection.

### Evaluation of intraoperative and postoperative adverse events

Record the duration of surgery and anesthesia, the total dose of opioids and fluids, bleeding and urine output. Adverse events recorded intraoperatively included bradycardia (< 40 beats/min), tachycardia (> 120 beats/min), hypertension (> 20% above baseline or systolic > 160 mmHg) and hypotension (> 20% below baseline or systolic < 80 mmHg). Assess for postoperative complications such as cerebrovascular events, heart failure, myocardial infarction and acute kidney injury.

### Statistical analysis

The Kolmogorov–Smirnov test is used to identify the normality of continuous variables. For continuous variables, the mean value ± Standard Deviation (SD) or the median value ± Interquartile Range (IQR) was reported according to the normality distribution. For categorical variables, the data were reported as percentage figures. The difference between the dexmedetomidine group and the control group was measured by unpaired T-test for normal distribution continuous variables, Mann–Whitney U test for abnormal distribution continuous variables, and χ^2^ test for categorical variables. IBM SPSS 22.0 software was used for statistical analysis, and P < 0.05 was considered as a difference of statistically significant.

## Results

### Study population

**Figure 1:**
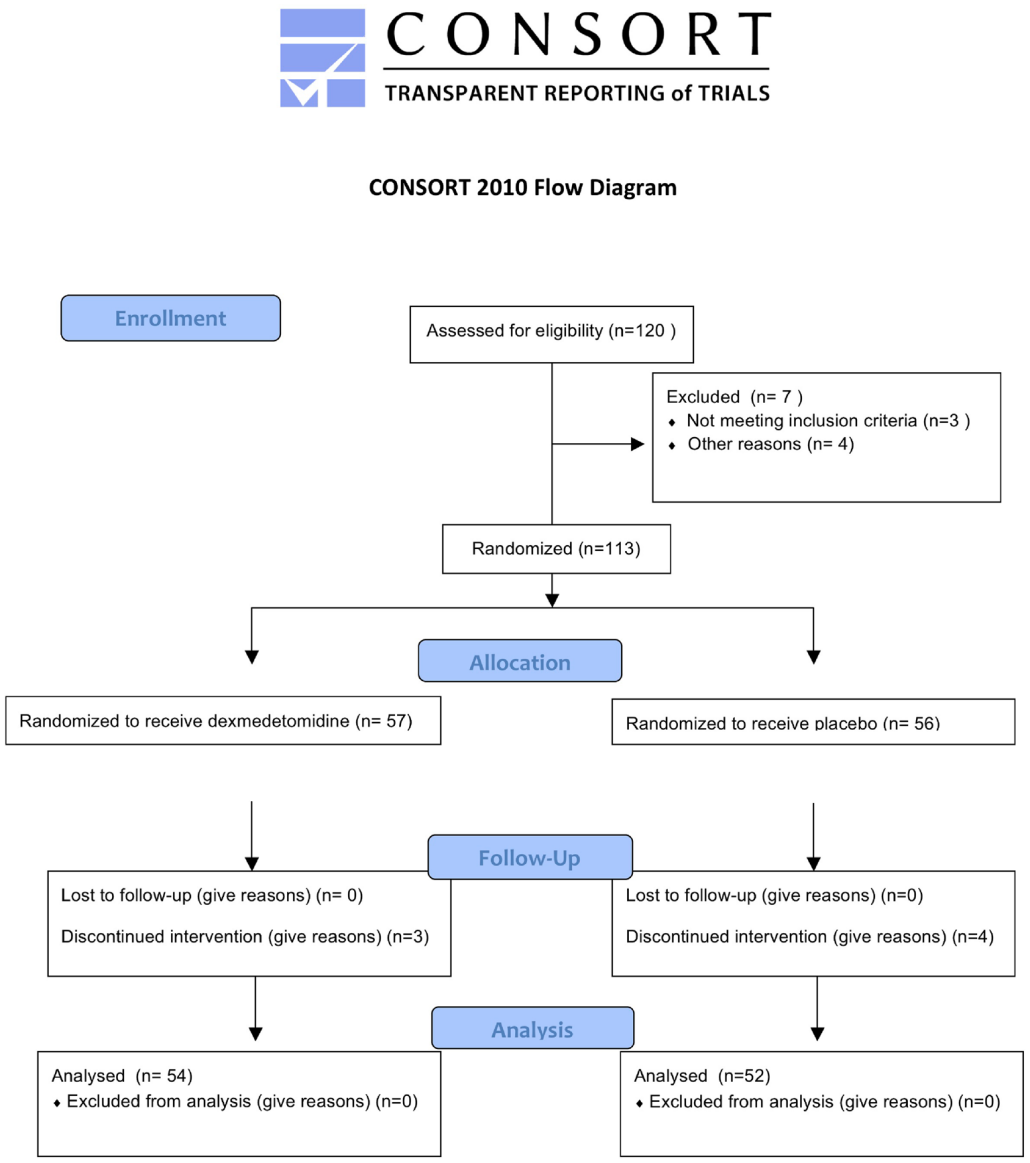
CONSORT flow diagram.

**Figure 2:**
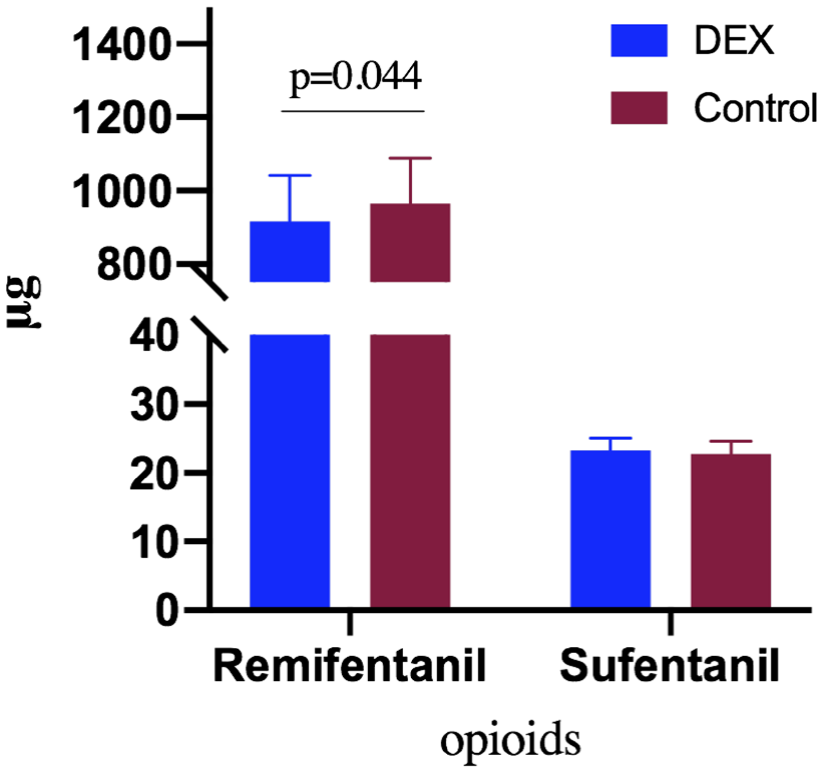
The intraoperative remifentanil consumption of the dexmedetomidine group was significantly lower than those of the control group. Meanwhile, intraoperative sufentanil consumption was similar. The dosages are presented as mean and standard deviation. *DEX* dexmedetomidine.

Of the 120 patients, 7 did not meet the inclusion criteria and the other 113 were randomized to receive dexmedetomidine (N = 57) and placebo (N = 56) (Fig. 1). Due to changes in surgical regimen, 7 patients were excluded. The remaining 106 patients (54 in dexmedetomidine group and 52 in placebo group) were included in the final analysis. In total, the following aspects of the two groups (dexmedetomidine vs placebo) were comparable at baseline: Age, BMI, ASA grading I and II, operation time, anesthesia time, hypertension, mellitus and intraoperative use of vasoactive (Table 1). There was little difference in the intraoperative blood loss, infusion volume, urine volume and the dose of long-acting opioid analgesic sufentanil and postoperative analgesic drug tramadol between the two groups, however, the dose of short-acting opioid analgesic remifentanil used in the dexmedetomidine group was significantly less than that of the placebo group (SD, μg, 916.09 [126.76] *vs* 965.56 [123.43], P = 0.044) (Fig. 2).

**Table 1:** Patient characteristics and baseline data.

Characteristic	Dexmedetomidine group	Control group	Z or χ^2^	P value
Total patients, no.	54	52	NA	NA
Age, mean (SD), year	46.89 (3.71)	46.71 (3.66)	0.248	0.805
BMI, mean (SD)	26.59 (2.13)	26.02 (2.19)	1.374	0.172
**ASA classification (n, %)**			1.608^&^	0.301
I	44 (81.5)	38 (73.1)		
II	10 (18.5)	14 (26.9)		
**Time, mean (SD), min**				
Surgical	76.83 (19.25)	74.13 (20.17)	0.705	0.482
Anesthetic	83.69 (20.84)	80.71 (21.90)	0.716	0.475
Hypertension (Y/N)	21/43	26/26	1.325^&^	0.250
Mellitus (Y/N)	12/42	12/40	0.011^&^	0.916
Use of vasoactive drugs (n, %)	6 (11.1)	9 (17.3)	0.837^&^	0.360
Tramadol (IQR), mg	0 (0–100)	0 (0–100)	− 1.310	0.190
Bleeding, ml^#^	57.79 (9.66)	57.28 (8.90)	0.282	0.778
Crystalloid solution, ml^#^	1087.41 (157.35)	1069.23 (146.08)	0.616	0.539
Urine output, ml^#^	365.74 (50.31)	363.46 (47.61)	0.239	0.812

*NA* not applicable, *ASA* American society of anesthesiologists, *BMI* body mass index, *SD* standard deviation, *n* number, *Y* yes, *N* no.

^#^Values were rounded to the nearest 10 mL.

^&^χ^2^ value.

### Primary outcome

The median time to first flatus in the dexmedetomidine group was significantly shorter than that in the control group (SD, 25.83 [4.18] vs 27.67 [3.77], P = 0.019) (Table 2).

**Table 2:** Clinical postoperative outcomes.

Characteristic	Dexmedetomidine group	Control group	t or χ^2^	P value
Total patients, no.	54	52	NA	NA
**Primary outcome**				
Time to first flatus, h	25.83 (4.18)	27.67 (3.77)	− 2.378	0.019
**Secondary outcomes**				
Time to first oral feeding, h	27.29 (4.40)	28.92 (3.82)	− 2.040	0.044
Time to first feces, h	59.82 (10.49)	63.89 (7.71)	− 2.270	0.025
Abdominal distension (n, %)	12 (22.2)	21 (40.4)	4.076^&^	0.044
PONV 24 h (n, %)	10 (18.5)	19 (36.5)	4.328^&^	0.037
PONV 48 h (n, %)	8 (14.3)	16 (30.8)	3.850^&^	0.050

*NA* not applicable, *PONV* postoperative nausea, and vomiting, *NRS* numeric rating scale, *h* hour, *n* number.

^&^χ^2^ value.

### Secondary outcomes

**Figure 3:**
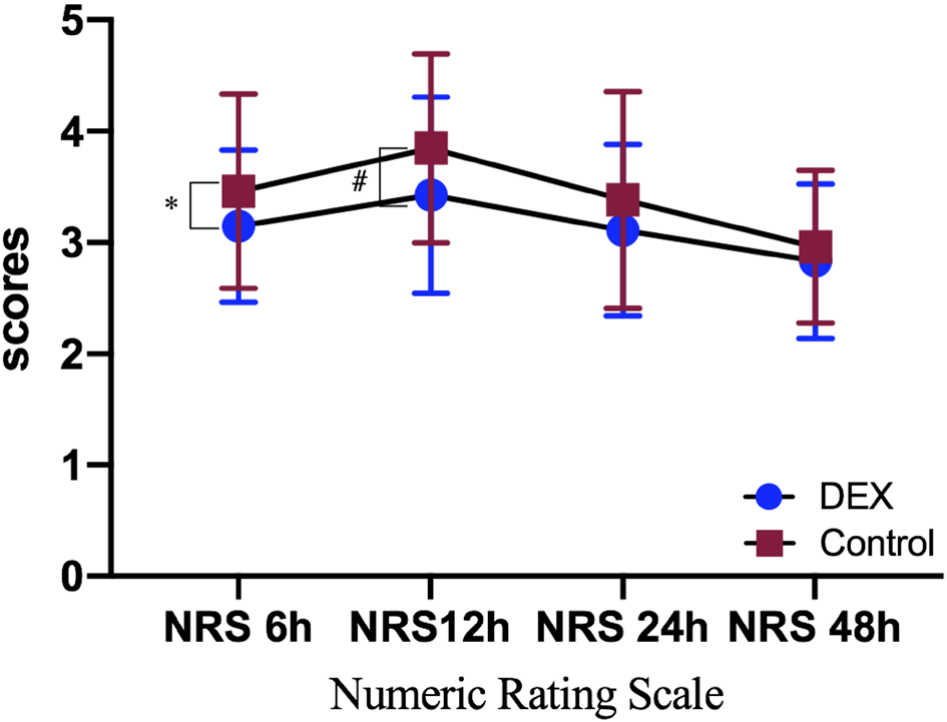
The postoperative pain scores of the dexmedetomidine group were significantly lower than those of the control group at 6 h and 12 h after surgery. The scores are presented as mean and standard deviation. *DEX* dexmedetomidine, *NRS* numeric rating scale. *P = 0.043, ^#^P = 0.014.

The following aspects of the two groups (dexmedetomidine vs placebo) were comparable: feeding time (SD, 27.29 [4.40] vs 28.92 [3.82], P = 0.044), the time to first defecation (SD, 59.82 [10.49] vs 63.89 [7.71], P = 0.025) (Table 2), the occurrence of flatulence (12 patients [18.5%] vs 21 patients [40.4%], P = 0.044), the incidence of PONV 24 h (10 patients [18.5%] vs 19 patients [36.5%], P = 0.037) and 48 h after surgery (8 patients [14.8%] vs 16 patients [30.8%], P = 0.05). The postoperative pain scores of the dexmedetomidine group were significantly lower than those of the control group at 6 h and 12 h after surgery (SD, 3.15[0.68] vs 3.46 [0.88] and 3.11 [0.77] vs 3.38 [0.97]), respectively, P < 0.05) (Fig. 3).

### Adverse events

The incidence of severe hypoxemia (1 patient [0.02%] vs 1 patient [0.02%]) for patients in the dexmedetomidine group and control group during the first 2 days after surgery were similar. The incidence of hypotension (6 [11.1%] vs 6 [11.5%]) and hypertension (8 [14.8%] vs 10 [19.2%]) requiring treatment were similar between the dexmedetomidine and control group, and the incidence of bradycardia (13 [24.1%] vs 5 [9.6%], P = 0.047) with a statistical significance (Table 3). None of the patients from each group had a post-operative stroke, myocardial infarction or heart failure.

**Table 3:** Adverse events.

Characteristic	Dexmedetomidine group	Control group	χ^2^	P value
Total patients, no.	54	52	NA	NA
Severe hypoxemia (n, %)	1 (1.9)	1 (1.9)	0.001	0.979
Intra-hypotension (n, %)	6 (11.1)	6 (11.5)	0.005	0.945
Intra-hypertension (n, %)	8 (14.8)	10 (19.2)	0.366	0.545
Intra-bradycardia (n, %)	13 (24.1)	5 (9.6)	3.928	0.047
Intra-tachycardia (n, %)	3 (5.6)	5 (9.6)	0.626	0.429

*NA* not applicable.

## Discussion

This randomized clinical trial investigated the effects of intraoperative intravenous dexmedetomidine on gastrointestinal function recovery in women undergoing laparoscopic hysteromyomectomy. The present study showed that dexmedetomidine administration reduced the time to first flatus, the time to first oral feeding and the time to first defecation, the intraoperative short-acting opioid requirement. Meanwhile, it alleviated pain score and abdominal distension, lowered PONV occurrence. Adverse reactions to dexmedetomidine are mainly limited to hemodynamic changes such as bradycardia, transient hypertension, and hypotension, which is a major side effect of dexmedetomidine^21^. In this study, there were no significant differences in these adverse events between the two groups, except improvable bradycardia.

The pathogenesis of POI remains unclear, which involves many factors including sympathetic and parasympathetic nerve regulation, inflammatory changes mediated by multiple pathways, dysregulation of the intestinal immune system, and the use of opioids for the management of postoperative pain, etc^22,23^. Especially, inflammation of the extramental muscular is one of the main mechanisms of POI^24^. Surgical intervention of the intestine activates innate immune cells located in the outer muscular layer, resulting in the release of inflammatory cytokines and chemokines and the increased expression of adhesion molecules on endothelial cells, thereby leading to the invasion of circulating leukocytes in the muscular externa^25,26^. As innate and adaptive inflammatory mediators, neutrophils are the first-line defensive immune cells that act against tissue damage and pathogen invasion through rapid mobilization, phagocytosis, intracellular killing, the release of antibacterial factors and neutrophils extracellular traps, later, dendritic cells, mast cells, and macrophages may also be involved^1,27,28^. Intestinal manipulation during abdominal hysterectomy results in the immediate release of the mast cell activation marker, trypsin, in peritoneal fluid, followed by an increase of pro-inflammatory cytokines IL-6 and IL-8^29^. Dissemination is an important feature of POI, which means that if only part of the intestine is handled or inflamed, the movement of the entire gastrointestinal tract will be impaired. The underlying mechanism may involve the activation of inhibitory neural pathways by inflammatory mediators. One theory is that help T (Th) cells respond to the inflammatory part and then disseminate to the whole gastrointestinal tract, which is also a potential mechanism for POI^24^. The nerve regulation of the intestinal is essential. Sympathetic/parasympathetic pathways to the gastrointestinal include three distinct reflexes: ultrashort reflexes confined within the bowel wall, short reflexes involving prevertebral ganglia, and long reflexes involving the spinal cord^30^. Through these reflexes, activated sympathetic nerves could increase the release of catecholamine, which inhibits postoperative gastrointestinal function by limiting intestinal smooth muscle contraction^31^. In animal models, stimulation of the parasympathetic nerve has been shown to reduce the level of tumor necrosis factor (TNF), relieve POI, and improve survival^32^. Therefore, although laparoscopic surgery significantly reduces the time to recovery of intestinal function, CO_2_ used to establish pneumoperitoneum and other factors can still directly or indirectly activate the sympathetic nerve, resulting in the increase of catecholamine level and the inhibition of postoperative gastrointestinal function recovery, which is also one of the main causes for postoperative intestinal paralysis^33,34^.

Dexmedetomidine is one of the latest studied anesthetic adjuncts and a highly selective α 2-adrenergic receptor agonist that by acting on the α 2-adrenergic receptors in the central nervous system, it reduces sympathetic activation and catecholamine release^35,36^. It protects organ function by reducing the release of neurotransmitters from sympathetic ganglion fibers, thereby reducing the production of relevant inflammatory factors, inhibiting oxidative stress, reducing reperfusion injury^37^ and dose-dependently inhibiting the production of neutrophil chemokines, CXCL1 and CXCL2^38^. Dexmedetomidine can directly affects cytokine-producing cells such as monocytes/macrophages and is associated with the reduction of perioperative norepinephrine level, thus, it may alleviate the reduced phagocytic capacity of neutrophils^39^. A meta-analysis indicated that the perioperative administered of dexmedetomidine as an adjunct to general anaesthesia had anti-inflammatory effects^40^. Xiang et al. confirmed that dexmedetomidine can increase the discharge frequency of the vagus nerve in rats and inhibit the release of inflammatory factors through a cholinergic anti-inflammatory pathway^41^. In a sheep model of septic shock, dexmedetomidine can improve intestinal microcirculation and reduce lactic acid production within, thereby improving survival rate^42^, suggesting that dexmedetomidine may improve the intestinal microcirculation perfusion of surgical patients. Besides, dexmedetomidine alleviates intestinal ischemia–reperfusion injury by inhibiting the toll-like receptor 4/MyD88 signaling pathway, which may be the mechanism by which it promotes gastrointestinal function recovery^43^. Yeh et al. used magnetic resonance imaging(MRI) determination of gadolinium in serum diamine method to assess the level of intestinal permeability, the results show that dexmedetomidine can reduce cell apoptosis in the intestinal mucous membrane, thereby protecting the intestinal permeability and barrier function^44^, which may also be the mechanism by which dexmedetomidine improves postoperative bowel function recovery. In general, dexmedetomidine reduces perioperative stress response, benefits anti-inflammatory status, regulates the intestinal immune system, and increases the discharge frequency of the parasympathetic nerve, which leads to the reduction in the incidence of postoperative ileus.

Previous studies have shown that dexmedetomidine can reduce the time to first flatus and improve the intestinal function recovery of the elderly patients undergoing intestinal surgery^45^. For patients undergoing laparoscopic colorectal cancer resection, intraoperative dexmedetomidine (1 μg kg^−1^ loading dose over 10 min, maintain with a dose of 0.3 μg kg^−1^ h^−1^) with a reduced time to the first flatus^46^. Likewise, infusion of 0.04 μg kg^−1^ h^−1^ dexmedetomidine is beneficial to the recovery of intestinal function in patients who underwent laparoscopic nephrectomy^47^. Patients undergoing lumbar fusion received dexmedetomidine (loading dose of 0.5 μg kg^−1^, infusion time over 15 min, maintain with a dose of 0.1 μg kg^−1^ h^−1^) or normal saline, with a short first fart time, less total dose of sufentanil in the dexmedetomidine group^48^. Identically, in our present study, a single dose of 0.5 μg kg^−1^ dexmedetomidine or the same volume of placebo (normal saline) was intravenously administered for 15 min, followed by continuous pumping of 0.2 μg kg^−1^ h^−1^ of corresponding drugs until 30 min before the end of laparoscopic hysteromyomectomy. The results showed a low dose of dexmedetomidine significantly promoted the recovery of gastrointestinal function. However, some studies have reported conflicting results. Compared with normal saline, dexmedetomidine 1 μg kg^−1^ for 20 min and 0.7 μg kg^−1^ h^−1^ for 190 min significantly inhibited gastric emptying and gastrointestinal transport in healthy subjects^49^. Studies in mice have shown that dexmedetomidine can inhibit gastrointestinal function in septic shock mice through Ca^2+^ response on intestinal glial cells^50,51^. One study suggested that dexmedetomidine does not affect the intestinal function of patients undergoing abdominal hysterectomy^17^. The possible explanations for these conflicting results are as follows: First, the pathophysiological state of patients’ understudies was different, such as normal volunteers. Second, the infusion speed, route of medication, and total volume of dexmedetomidine used among studies were different. Third, the gut may be severely disturbed in severely immunosuppressed septic shock mice. The dose of dexmedetomidine used was higher in studies suggesting an inhibition effect on intestinal function, while low-dose dexmedetomidine could yield benefits.

POI is a common secondary effect of opioids and has significant clinical and economic impacts. Opioids are widely used for perioperative analgesia. Therefore, to prevent POI, the use of opioids should be minimized^52^. Remifentanil is an ultra-short-acting opioid that is rapidly metabolized in the plasma and tissue by nonspecific esterases to an inactive metabolite. It has a very brief elimination half-life, with a context-sensitive half-life of only 3 min, independent of the duration of infusion^53^. Therefore, the opioid remifentanil has been considered independent risk factor development of POI, previously^54^. Consistent with previous reports^55^, there is a significant difference in the dose of intraoperative remifentanil. Apart from decreasing the use of remifentanil, intraoperative administration of dexmedetomidine reduced the postoperative pain scores of patients in this study, which may have beneficial effects on the postoperative recovery of gastrointestinal function. A meta-analysis showed that dexmedetomidine administration was associated with a lower rest pain score at 2 h post-operatively, compared with remifentanil^55^. In patients undergoing robotic urological surgery, intravenous dexmedetomidine has equal analgesic efficacy as fentanyl and can be used as the sole analgesic agent^56^. Peripheral action μ-opioid receptor antagonists (PAMORAs) can block the opioid action on the gastrointestinal tract while maintaining its analgesic action on the central nervous system and is effective for the treatment of opioid-related bowel dysfunction and POI^57,58^. Literature suggests that the key to the effect of dexmedetomidine on perioperative intestinal function lies in its ability to reduce the dosage of opioids^59,60^. Moreover, the 2018 enhanced recovery after surgery (ERAS) guidelines strongly recommend dexmedetomidine as part of multimodal analgesia and reduction in opioid consumption^61^. However, the intraoperative remifentanil infusion may be beneficial for the recovery of postoperative intestinal function due to its prevention of intraoperative intestinal ischemia–reperfusion injury, although none of them was in humans^62–64^, indicating remifentanil has a complex effect on postoperative ileus. More studies are needed to confirm the pharmacological effects of remifentanil on postoperative ileus.

## Conclusion

In patients undergoing laparoscopic hysteromyomectomy, intraoperative intravenous administration of dexmedetomidine significantly reduced the time to first flatus, first oral feeding and first defecation. Dexmedetomidine may be considered for improving the postoperative recovery of intestinal function in patients undergoing laparoscopic hysteromyomectomy.

### Limitations

The study has several limitations. First, our study was conducted in a single-center, the conclusion of which needs to be confirmed by multiple centers. Second, the serum concentration of dexmedetomidine was not measured, thus the exact serum concentration of dexmedetomidine in different patients was not clear. As discussed, the mechanism of action of dexmedetomidine may be related to its blood concentration, therefore, further studies are needed to explore the relationship between the dose of dexmedetomidine and gastrointestinal function. Third, the pathophysiology of perioperative intestinal dysfunction and intestinal ileus has not been fully elucidated, nor has the relevant mechanism analyzed. More studies are needed to explore the underlying mechanisms and explore appropriate interventions to remedy POI. Last but not least, as the variation in intraoperative remifentanil consumption, this may be a crucial influence factor affect postoperative intestinal function and further research is required. These limitations should be avoided in our future studies.

## Data Availability

The datasets used and analyzed during the current study are available from the corresponding author on reasonable request.
